# RX-3117 (Fluorocyclopentenyl-Cytosine)-Mediated Down-Regulation of DNA Methyltransferase 1 Leads to Protein Expression of Tumor-Suppressor Genes and Increased Functionality of the Proton-Coupled Folate Carrier

**DOI:** 10.3390/ijms21082717

**Published:** 2020-04-14

**Authors:** Dzjemma Sarkisjan, Joris R. Julsing, Btissame El Hassouni, Richard J. Honeywell, Ietje Kathmann, Larry H. Matherly, Young B. Lee, Deog J. Kim, Godefridus J. Peters

**Affiliations:** 1 Laboratory Medical Oncology, Amsterdam UMC, location VU University Medical Center, 1081 HV Amsterdam, The Netherlands; d.sarkisjan@amsterdamumc.nl (D.S.); jr.julsing@vumc.nl (J.R.J.); B.elhassouni@amsterdamumc.nl (B.E.H.); r.honeywell@amsterdamumc.nl (R.J.H.); i.kathmann@vumc.nl (I.K.); 2 Department of Oncology, Wayne State University School of Medicine, Detroit, and Molecular Therapeutics Program, Barbara Ann Karmanos Cancer Institute, Detroit, MI 48201-1976, USA; matherly@karmanos.org; 3 Rexahn Pharmaceuticals, Inc., Rockville, MD 20850, USA; yolee@hotmail.com (Y.B.L.); kimdj@rexahn.com (D.J.K.); 4 Department of Biochemistry, Medical University of Gdansk, 80-210 Gdansk, Poland

**Keywords:** RX-3117, 5-aza-2′-deoxycytidine, hypomethylation, proton-coupled folate receptor, methotrexate

## Abstract

(1) Background: RX-3117 (fluorocyclopentenyl-cytosine) is a cytidine analog that inhibits DNA methyltransferase 1 (DNMT1). We investigated the mechanism and potential of RX-3117 as a demethylating agent in several in vitro models. (2) Methods: we used western blotting to measure expression of several proteins known to be down-regulated by DNA methylation: O^6^-methylguanine-DNA methyltransferase (MGMT) and the tumor-suppressor genes, p16 and E-cadherin. Transport of methotrexate (MTX) mediated by the proton-coupled folate transporter (PCFT) was used as a functional assay. (3) Results: RX-3117 treatment decreased total DNA-cytosine-methylation in A549 non-small cell lung cancer (NSCLC) cells, and induced protein expression of MGMT, p16 and E-cadherin in A549 and SW1573 NSCLC cells. Leukemic CCRF-CEM cells and the MTX-resistant variant (CEM/MTX, with a deficient reduced folate carrier) have a very low expression of PCFT due to promoter hypermethylation. In CEM/MTX cells, pre-treatment with RX-3117 increased PCFT-mediated MTX uptake 8-fold, and in CEM cells 4-fold. With the reference hypomethylating agent 5-aza-2′-deoxycytidine similar values were obtained. RX-3117 also increased *PCFT* gene expression and PCFT protein. (4) Conclusion: RX-3117 down-regulates DNMT1, leading to hypomethylation of DNA. From the increased protein expression of tumor-suppressor genes and functional activation of PCFT, we concluded that RX-3117 might have induced hypomethylation of the promotor.

## 1. Introduction

**Figure 1:**
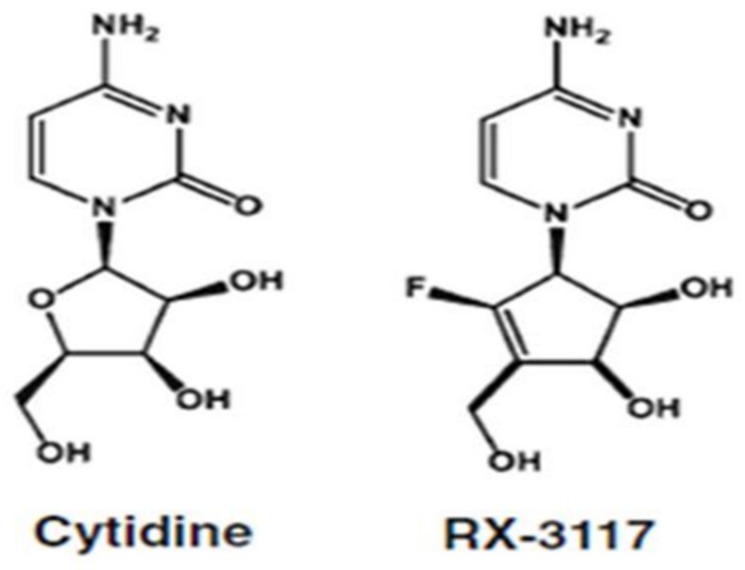
Chemical structure of cytidine and the cytidine analog RX-3117.

RX-3117 is a novel cytidine analog modified in the sugar ring moiety [1] (Figure 1), which has shown promising anti-tumor activity against gemcitabine resistant xenografts from different pathologies [2] and is currently in clinical trial (NCT02030067) [3]. RX-3117 was shown to down-regulate DNA methyl transferase 1 (DNMT1) [1,3,4,5,6]. The pharmacological consequences of this inhibition, however, have not been identified. DNMT1 acts as a maintenance methylation enzyme for newly synthesized DNA and ensures methylation patterns in newly dividing cells, using already existing hemi-methylation patterns [7]. Cytidine rich regions of the DNA are substrates for DNMT1, so-called CpG islands [8]. Promoters of genes are enriched with CpG islands and methylation of gene promoters has a silencing effect on gene transcription [5]. Many inhibitors of DNMT1 are base-modified cytidine analogs such as 5-aza-cytidine (AzaC) and 2′-deoxy-5-aza-cytidine (DAC) [9,10] and are registered for treatment of hematological malignancies [5]. RX-3117 is a cytidine analog modified in the sugar.

Hyper-methylation of tumor-suppressor gene (TSG) promoters is known to have implications in cancer progression [11] and is of therapeutic interest, as hypermethylation of TSG promoters causes suppression of gene transcription and silencing of TSGs [9] whereas CpG demethylation results in TSG transcriptional activation [12].

To study whether down-regulation of DNMT1 by RX-3117 leads to demethylation of DNA at a functional level, we determined whether protein expression of genes down-regulated by extensive DNA methylation can be reactivated. For this purpose, we used the non-small cell lung cancer (NSCLC) cell lines SW1573 and A549, each known to have specific aberration in certain TSGs. To investigate whether RX-3117 can also reactivate functional activity of a gene we focused on the proton-coupled folate transporter (PCFT), which is a transmembrane folate and anti-folate transporter [13]. PCFT expression is highly regulated by promoter methylation [14,15,16,17] and, due to extensive methylation of the gene promoter, expression is very low [14,18], leading to a loss of transporter function [16]. A high level of promoter methylation and a low PCFT expression were associated with a poor efficacy of pemetrexed (PMX)-based treatment of malignant mesothelioma [18]. Both PMX and the widely used classical anti-folate methotrexate (4-amino-10-methylpteroyl-glutamic acid, MTX) [19,20] are excellent substrates for PCFT at a low pH environment [13].

PCFT is one of the three major cellular membrane folate transporters, in addition to the reduced folate carrier (RFC) and the folate receptor (FR) α [20,21,22]. FRα is a high affinity, low capacity transmembrane transporter for oxidized folates such as folic acid [23], whereas RFC and PCFT have a much higher transmembrane transport capacity depending on the substrate. The major difference between RFC and PCFT is the optimal external pH at which they exert their function, PCFT being most efficient at an acidic pH of 5.5 (a pH at which RFC is not functional), while RFC has an optimal pH of 7.4 (where PCFT is active, but less effective) [13,14,16,20,22]. These membrane transporters also have an excellent affinity for anti-folate drugs extensively used for cancer treatment, such as PMX and MTX [22].

The CCRF-CEM (CEM) leukemia cell line shows a high innate methylation of the PCFT gene promoter and consequently shows a low PCFT expression [14,18]. CEM cells have been used as a model to determine whether promoter hypomethylation increased the uptake of MTX and PMX [14,18]. Since treatment with DAC leads to hypomethylation and increased uptake of both PMX and MTX in CEM parental cells and its MTX-resistant variant [18], CEM/MTX, we used these cells to determine whether RX-3117 treatment would reactivate PCFT gene expression and mediated MTX uptake.

## 2. Results

### 2.1. Targeting DNA Methylation

A549 and SW1573 NSCLC cells, show hypermethylation of the promoters of several genes including TSGs [23]. Since hypermethylation of TSGs is cell line dependent we chose A549 cells to study the effect of RX-3117 on protein expression of O-6-methylguanine-DNA methyltransferase (MGMT) and E-cadherin, while SW1573 cells were chosen to study the effect on p16. Earlier we demonstrated a concentration and time-dependent down-regulation of DNMT1 in cell lines with various histological backgrounds (NSCLC, ovarian cancer, pancreatic cancer, breast cancer) [1,3,4]. 

**Figure 2:**
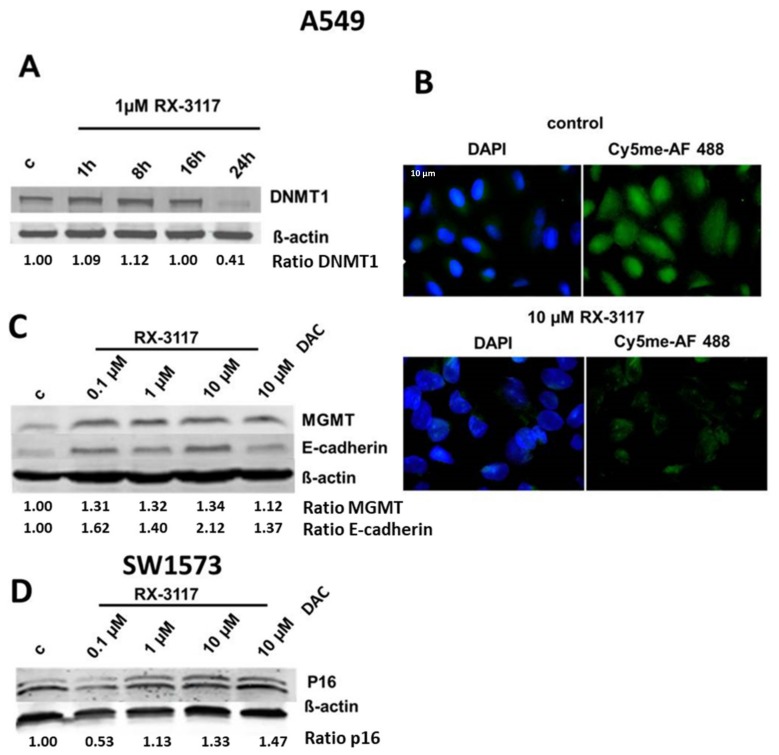
The effect of RX-3117 mediated DNMT1 down-regulation on DNA methylation and expression of target proteins (ratio to β-actin) in A549 and SW1573 NSCLC cells. **A**: Time dependency of DNMT1 down-regulation. Western blot analysis of cell lysates after exposure to 1 µM RX-3117 after 1, 8, 16, and 24 h; β-actin was an internal loading control. **B**: Confocal microscopy pictures of A549 cells stained for the nucleus with DAPI (blue) and for total DNA methylation with 5mC Alexa Fluor-488 (green). The upper panel shows a control of untreated cells, the lower panel shows cells treated for 24 h with 10 µM RX-3117. Staining was predominantly nuclear. **C**: Western blot analysis of the TSG encoded proteins, MGMT and E-cadherin upon exposure to increasing concentrations of RX-3117 (0.1, 1 and 10 µM). As a positive control 10 µM of DAC was used and an internal control of β-actin was included. **D**: Western blot analysis of the TSG encoded protein p16 in SW1573 cells upon exposure to increasing concentrations of RX-3117 (0.1, 1 and 10 µM). The lower band under the p16 protein band, however, is a non-specific band. As a positive control 10 µM of DAC was used and an internal control of β-actin was included. This concentration of DAC was chosen based on its IC50 value in these cell lines. The blots are original, representative for one out of 2–3 separate experiments.

SW1573 cells require a higher concentration of RX-3117 than A549 cells [4]. Since DNMT1 down-regulation is time-dependent, as shown for A549 cells (Figure 2A), we chose a 24 h exposure to determine the effect of RX-3117 on total hypomethylation and protein expression of various TSGs (Figure 2B–D).

Treatment with RX-3117 led to a decreased global DNA hypomethylation with a decreased nuclear staining. Assuming that these conditions would also lead to specific promoter hypomethylation, we exposed A549 cells to increasing concentrations of RX-3117 and protein expression of MGMT and E-cadherin were evaluated. DAC, a known demethylating agent, was used as a positive control in this experiment. Even 0.1 µM RX-3117 treatment for 24 hr substantially increased the levels of MGMT and E-cadherin proteins in the sensitive A549 cells; the increase was more pronounced for E-cadherin at 10 μM RX-3117 compared to DAC (Figure 2 C). Treatment with DAC also led to an increase in MGMT protein expression as seen with RX-3117 (Figure 2C). To determine the effect of RX-3117 treatment on p16 expression we exposed SW1573 cells to 1-10 µM RX-3117, which led to re-activation of suppressed p16 (Figure 2D).

These results demonstrate that RX-3117 treatment leads to DNMT1 down-regulation, DNA hypomethylation, and increased levels of proteins encoded by oncogenic genes known to be controlled by DNA promoter methylation.

### 2.2. Modulation of Methotrexate Uptake by RX-3117

We previously showed that a high PCFT expression is associated with a longer survival of patients treated with the anti-folate PMX and demonstrated that treatment with the hypomethylating drug DAC increased PMX uptake in mesothelioma cells [18]. To provide a functional readout of increased gene expression resulting from RX-3117-mediated hypomethylation, we examined whether activation of the PCFT gene expression would lead to an increase in the PCFT transport. To have a clean model system, we used CCRF-CEM leukemia cells, known to have a highly methylated PCFT promotor and an almost absent (undetectable) protein expression [14]. To exclude the contribution of the other important folate transporter RFC [18], we also used CEM/MTX cells which are resistant to MTX because of a deficiency in RFC-mediated transport [24]. As a transport substrate, we used radiolabeled MTX.

Since PCFT-mediated transport is optimal at pH 5.5 with a modest uptake at pH 7.4, we first determined MTX uptake at the physiological pH 7.4 as well as pH 5.5 in both CCRF-CEM parental cells and the RFC-deficient CEM/MTX cells (Table 1). At pH 7.4, MTX uptake in CEM/MTX was 93-fold lower than in parental CCRF-CEM cells, but at pH 5.5, this was only 2.2-fold (Table 1). Competitive inhibition with L-LV at pH 7.4 led to a 60-fold reduction of RFC-mediated MTX uptake in parental cells, but only 3-fold in CEM/MTX cells. The remaining MTX uptake is mediated by PCFT in both cell lines. Exposure to RX-3117 did not affect MTX uptake at pH 5.5. Only a slight increase was observed in CEM cells, but not in CEM/MTX cells. No effect of DAC was observed as well. Apparently the PCFT-mediated uptake was too high to measure an additional effect of either DAC or RX-3117. Since PCFT-mediated uptake at pH 7.4 would be much lower we expected that modulation (i.e., an increase) of PCFT-mediated uptake would be easier to detect (Table 1).

**Figure 3:**
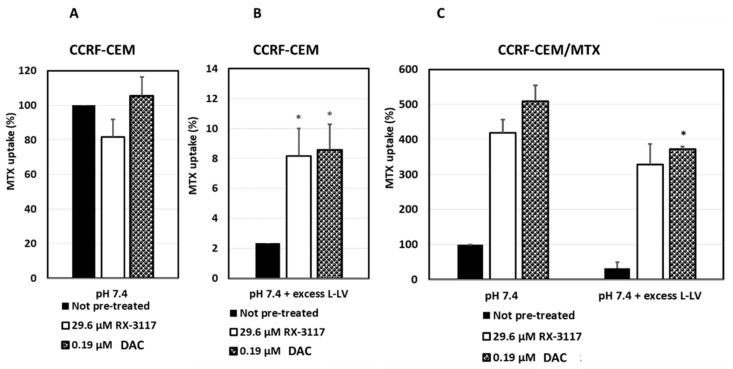
MTX uptake in CEM and CEM/MTX cell lines. Three conditions were used for CEM and CEM/MTX cells: control and 24 h pre-treatment with 26.9 µM RX-3117 or 0.19 µM DAC. Values are means ± SEM of three separate experiments. **A**: uptake of MTX in CEM cells with the uptake in untreated cells for each experiment normalized at 100%. **B**: uptake of MTX in CEM cells when RFC was completely blocked due to the addition of excess 1 μM L-LV. **C**: the left 3 bars show uptake of MTX in CEM/MTX cells, with the uptake in untreated cells for each experiment normalized to 100%. The right 3 bars show the uptake of MTX in CEM/MTX cells when residual RFC is blocked due to excess L-LV. *, significantly different from control untreated cells at the level of *p* < 0.01.

In CCRF-CEM cells, treatment with RX-3117, DAC or AzaC at their IC50 concentrations [4] did not affect MTX uptake at pH 7.4 (Figure 3A). However, when RFC was blocked by using an excess of L-LV, PCFT-mediated uptake of MTX was significantly higher in RX-3117 and DAC treated CCRF-CEM cells compared to control cells (Figure 3B). Also, AzaC treatment showed an almost 4-fold higher MTX uptake but this was not significant.

To exclude RFC-mediated uptake, we used CEM/MTX cells, which are almost completely RFC-deficient. RX-3117 increased transport of MTX into the cells about 4-fold and DAC about 5-fold (Figure 3C). Blocking the remaining RFC with l-LV increased this effect (Figure 3C, right part graph).

### 2.3. Re-Activation of PCFT by RX-3117

**Figure 4:**
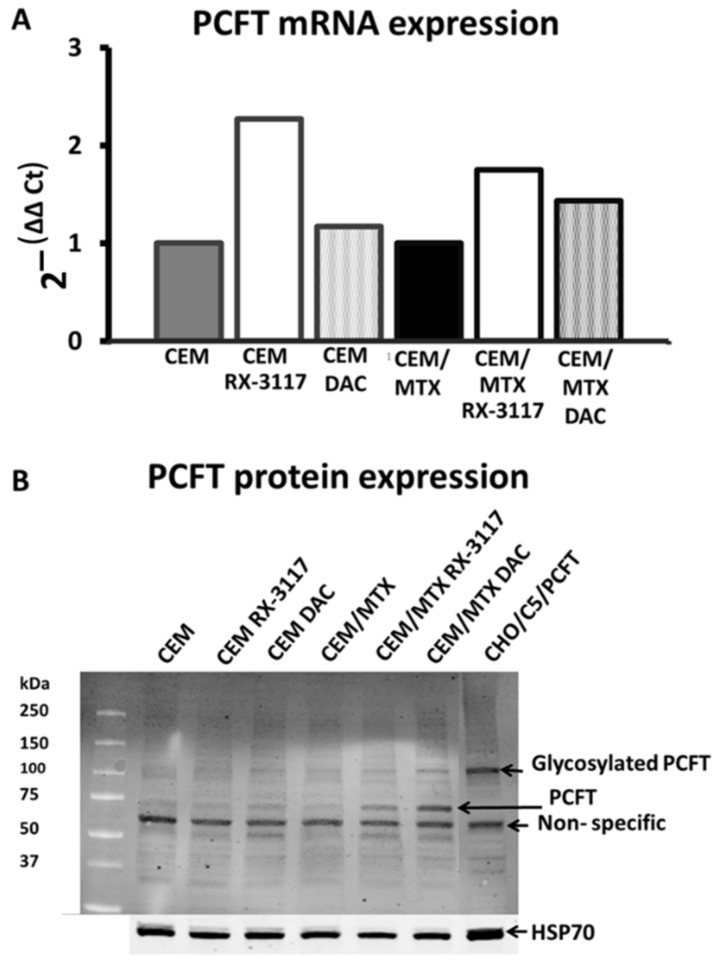
Gene and protein expression of PCFT in CEM and CEM/MTX cell lines after treatment with RX-3117 and DAC. **A**: RT-PCR data of PCFT gene expression normalized to beta-actin gene expression in CEM or CEM/MTX cells, non-treated, 24 h pre-treatment with 29.6 µM RX-3117 or 0.19 µM DAC **B**: Western blot data of PCFT protein expression in non-treated and after 24 h pre-treatment with 29.6 µM RX-3117 or 0.19 µM DAC. Loading control of the membrane compartment is HSP70 protein.

To demonstrate that the RX-3117-mediated increase in MTX uptake was indeed related to increased expression of PCFT gene and protein levels, we performed real-time PCR and western blotting, respectively (Figure 4). Indeed RX-3117 and DAC pre-treatment increased PCFT gene expression levels, both in CEM and CEM/MTX cells (Figure 4A). Since PCFT is a membrane associated protein we isolated the cellular membranes to evaluate the expression of PCFT. As expected in both CEM and CEM/MTX cells, PCFT protein expression was hardly detectable (Figure 4B). The CHO/C5/PCFT cells with an overexpression of PCFT were used to identify a positive PCFT band. These cells showed a high expression of glycosylated PCFT, but the CEM cells did not show any glycosylated PCFT at all. However, treatment with either RX-3117 or DAC resulted in appearance of PCFT protein at around 75 kDa, the expected MW, and of glycosylated PCFT at 100 kDa, which was more clearly visible in the CEM-MTX cells. Apparently the time-span might be too short to allow a high PCFT glycosylation in these purified membranes. We also observed a non-specific band around 60 kDa.

## 3. Discussion

In this paper, we demonstrate that RX-3117-mediated down-regulation of DNMT1 is associated with an increased protein expression of several silenced TSG such as MGMT, E-cadherin, and p16. Moreover, we demonstrate that RX-3117 treatment can reactivate functionality of PCFT, which was earlier shown to be caused by promoter methylation.

MGMT is an enzyme that plays a role in the DNA repair [25]. Methylation of the MGMT promoter is a favorable predictive factor in the treatment of glioma patients with temozolomide [26]. We studied protein expression of MGMT in A549 cells because this gene was known to be silenced in A549 cells; our data indeed show that RX-3117 treatment increased MGMT protein expression similar to the effect of the epigenetic modulator DAC. Although we did not measure promoter methylation, our data are in line with a hypomethylation induced increased expression of MGMT.

The product of the TSG E-cadherin is an extracellular receptor that mediates cell-cell interactions [27,28]. Loss of E-cadherin function is thought to be correlated with cancer progression by increasing the proliferation, invasion and metastasis [29,30]. Therefore, hypomethylation of the E-cadherin gene may increase the expression and inhibit cancer progression. Since RX-3117 treatment increased E-cadherin protein expression, the RX-3117-mediated growth inhibition may be related to E-cadherin stimulation. P16 regulates the cell cycle progression and is important for suppression in the formation of different cancer types [31,32]. Re-expression of p16 protein, as seen with RX-3117 treatment, may normalize cell cycle progression. Since DNMT1 expression is cell cycle regulated, this raises the question whether RX-3117 induced DNMT1 down-regulation might be a cell cycle effect. Indeed RX-3117 induces some cell cycle proteins (e.g., CHK2 and cdc25), with an arrest in the S and G2M phase), [33] but whether this is related to DNMT1 down-regulation is unlikely because of the different time-span. Altogether re-activation of TSGs may contribute to the elimination of tumor cells, by inhibition of tumor growth, invasion, and controlling metastasis. Our findings indicate that RX-3117 might have activity in tumors with silenced TSGs.

Earlier we demonstrated the importance of promotor methylation for PCFT in mesothelioma [18]. PMX is also first line treatment for patients with non-squamous NSCLC not eligible for treatment with one of the tyrosine kinase inhibitors such as erlotinib, gefitinib, or crizotinib [34]. Therefore, we investigated whether treatment with RX-3117 would be able to modulate PCFT protein and gene expression. For this purpose we chose a model system, previously well characterized for uptake and sensitivity to various antifolates including MTX and PMX [35]: CEM-CCRF cells and its MTX-resistant variant CEM/MTX, which has a deficient RFC expression, with mutations in the hotspot area of the *RFC* gene [21]. The cell line was previously characterized by an extensive methylation of the PCFT promotor which could be modulated by DAC [14], and was therefore considered to be an ideal target to validate whether RX-3117 treatment would have a functional effect with possible relevant effects for treatment of either mesothelioma or non-squamous NSCLC. RX-3117 induced PCFT functionality (increased gene and protein expression and increased transmembrane transport) which is likely the result of hypomethylation of the PCFT promoter as was also seen in earlier studies with DAC, which was included as a positive control in our studies.

In addition, this study underlines the relevance of DNA methylation as a potential biomarker for RX-3117′s anti-tumor activity. However, RX-3117 is also known to cause DNA damage by its incorporation into DNA [4,33]. Usually the induction of DNA damage by a nucleoside analog takes more time, so that the effects on DNA methylation may occur earlier then the DNA damage. Even though the exact mechanism of DNMT1 down-regulation is not clear yet, our study provides sufficient evidence to explore DNMT1 expression and DNA methylation as potential biomarkers for the efficacy of RX-3117. For instance, LINE-1 promoter methylation was observed in plasma of patients with advanced solid tumors, in attempts to use the promoter methylation of this gene as a biomarker for demethylating agent activity [36]. It is obvious that cancer treatment can be improved with the use of validated predictive biomarkers for the activity of a demethylating compound. Validation of biomarkers for RX-3117 activity includes a transporter, such as hENT or the activation enzyme UCK2, but DNMT1 or DNA methylation are worthwhile additions to clinical studies with this compound. A suitable source would be either circulating DNA [37] or circulating tumor cells [38]. Since all FDA approved hypomethylating agents are limited to leukemia, there is room for a novel demethylating agent that would work in solid tumors and would have less toxic side effects [39].

## 4. Materials and Methods

### 4.1. Cell Culture

The NSCLC cell lines A549 and SW1573 [6], were cultured in Dulbecco’s Modified Eagle Medium (DMEM) (Lonza, Verviers, Belgium) supplemented with 10% fetal bovine serum and 20 mM HEPES in T-25 flasks (Greiner Bio-One, Alphen aan de Rijn, the Netherlands). The leukemic cell lines CCRF-CEM (lymphoblastic leukemia) and its methotrexate-resistant variant CCRF-CEM/MTX (deficient in RFC) were cultured in RPMI1640 medium (Lonza, Verviers, Belgium) supplemented with 10% fetal bovine serum and 20 mM HEPES [24]. Cells were maintained in an experimental growth phase for all experiments and were tested negative for mycoplasma periodically every three months with the MycoAlert Mycoplasma Detection Kit (Cat no. LO LT07-705, Westburg, Leusden, The Netherlands).

### 4.2. Confocal Microscopy

Confocal microscopy experiments were performed to visualize the methylation of total DNA in A549 cells after 24–48 h treatment with 10 µM RX-3117. Cells were kept in exponential growth as indicated above and were imaged with a Zeiss Axiovert 200 Marianas inverted microscope (ZEISS) equipped with a motorized stage (stepper-motor *z*-axis increments, 0.1 μm), multiple fluorescence (FITC filter for 5meC and DAPI filter for nuclear stain) and a 63× oil immersion objective. Image acquisition and analysis were carried out under full software control (SlideBook 5.0.0.18; Intelligent Imaging Innovations, Göttingen, Germany).

### 4.3. Protein Expression

A549 cells were exposed to 1 µM RX-3117 at different time points: 1, 8, 16, and 24 h. Next, the total cell lysates were used to examine DNMT1 protein expression. PCFT, DNMT1, MGMT, p16, and E-cadherin protein expression were analyzed by western blots essentially as described earlier [4]. Briefly, cells were lysed using cell lysis buffer (Cell Signaling, Danvers, MA, USA), containing 4% protease inhibitor cocktail (Roche Diagnostics, Mannheim, Germany) on ice for 30 min and centrifuged for 10 min at 4 °C at 14,000 rpm. The Bio-Rad assay was used to determine the protein amount in the supernatant, as described earlier [4]. The membrane/organelle fraction of the cells was isolated using a protein extraction kit and handled according to manufacturer’s protocol (cat. # 539790; Sigma, St. Louis, MO, USA), with HSP70 as a loading control. The following antibodies were used: human PCFT (1: 1000) [18], HSP70 (1: 1000), β-actin (1: 10000, Sigma, St. Louis, MO, USA) and DNMT1 (1: 1000, #5032, Cell Signaling Technology, Inc., Danvers, MA, USA). Antibodies were diluted in a mixture of Rockland buffer (Rockland Inc, Philadelphia, PA, USA) and phosphate-buffered saline (PBS) supplemented with 0.05% Tween 20. Proteins were separated in 20% SDS-PAGE and transferred to a polyvinylidene difluoride (PVDF) membrane. For a fluorescent signal, goat anti-mouse InfraRedDye and goat anti-rabbit InfraRedDye secondary antibodies were used. Proteins were detected by an Odyssey InfraRed Imager (Li-COR Bioscience, Lincoln, NE, USA). The western blot images were quantified using ImageJ (National Institutes of Health, Bethesda, MD, USA) [40], by measuring the mean grey value of the inverted images. The background corrected values are given as target/housekeeping gene ratio relative to the control.

### 4.4. Modulation of Transport

To determine whether RX-3117 up-regulates PCFT, we measured the uptake of tritium (^3^H) labelled MTX in cells, as described earlier [24]. MTX uptake was determined at pH 5.5 and 7.4, the optimal pH for PCFT and RFC, respectively, which were competitively inhibited by addition of folic acid (FA) and l-leucovorin (L-LV), respectively. Uptake of MTX was performed in both CCRF-CEM and the RFC-deficient CCRF-CEM/MTX cell lines exposed to RX-3117 using AzaC and DAC as positive controls. Each condition required a minimum of 10 × 10^6^ cells. Twenty-four hours (h) before the uptake experiment, the cells were treated with either RX-3117, DAC, or no drug. The required number of cells was centrifuged at 1500× *g* for 5 min at room temperature and washed in 10 mL of HEPES-buffered saline (HBS) at pH 5.5 or 7.4 at room temperature. The cells were centrifuged at 1500 *g* for 5 min at room temperature and re-suspended in 1.1 mL per uptake condition in HBS at 37 °C and placed in a water bath of the same temperature. For each condition, one ml of cell suspension was added to a tube containing 10 μL of a 200 μM [^3^H]MTX at 37 °C and the tubes were continuously shaken for 3 min, after which uptake was stopped by adding 10 mL of ice-cold HBS with the appropriate pH. The cells were centrifuged at 1200 rpm for 5 min at 4 °C and subsequently re-suspended in 1 mL of 4 °C HBS (appropriate pH), after which 10 mL of ice-cold HBS (appropriate pH) was added and once more centrifuged at 1200 rpm for 5 min at 4 °C. Finally, the resulting pellets were dissolved in 150 μL of distilled water and transferred to 5.5 mL scintillation vials filled with 5 mL of scintillation fluid (Optiphase III), and counted 5 min for radioactivity in a scintillation counter.

### 4.5. PCFT Gene Expression

mRNAs from the same cell pellets (as for the drug uptake) were extracted and cDNAs were generated for gene expression analysis by RT-PCR. RNA isolation and cDNA synthesis were performed as described earlier [6]. Briefly, cells were washed and treated with TRIzol (Life Technologies, Bleiswijk, the Netherlands). RNA was extracted, washed after which the yield was determined by using a Nanodrop ND-1000 (Thermo Fisher Scientific, Wilmington, DE, USA). Synthesis of cDNA from an RNA sample was performed as described earlier [6] using reverse transcriptase (DyNAmo cDNA Synthesis kit; Thermo Fisher Scientific, Landsmeer, the Netherlands). Briefly, mRNA levels were determined by Taqman^®^ quantitative real-time PCR using a SDS7500 sequence detection system (Applied Biosystems, Foster City, CA, USA) (Applied Biosystems, Foster City, CA, USA). As a reference gene we used ß-actin with the following primer sets:

β-actin: Forward: TCACCCACACTGTGCCCATCTACGA

β-actin: Reverse: CAGCGGAACCGCTCATTGCCAATGG

β-actin: Primer: ATG CCCTCCCCCATGCCATCCTGCGT

The PCR reactions for PCFT were performed using the Hs00560565_m1 Assay-on-Demand product (Applied Biosystems, Foster City, CA, USA) [18]. For quantification of the fold increase in gene expression we used the ΔΔCt method and results are expressed as 2^−(ΔΔCt)^.

### 4.6. Statistical Analysis

For statistical analysis, Microsoft Excel 2010 was used. Student t-tests were performed to analyze statistical significance for the MTX uptake in cells. *p* values ˂ 0.05 were considered to be statistically significant.

## 5. Conclusions

The novel cytidine analog RX-3117 is a potent inhibitor of DNMT1. RX-3117 treatment leads to DNA hypomethylation and overexpression of proteins, such MGMT, E-cadherin, and p16, normally down-regulated by promoter hypermethylation. The effects were similar to that of DAC, which was included as a positive control for hypomethylation. PCFT is a membrane folate transporter highly regulated by promotor methylation. RX-3117 treatment led to an increase of PCFT-mediated MTX transport associated with increased PCFT gene and protein expression.

## Figures and Tables

**Table 1:** MTX uptake at different pH in CEM and RFC-deficient CEM/MTX cells.

External pH Level	CEM	CEM/MTX
Addition	MTX uptake (pmol/min/1 × 10^7^ cells)	
pH 5.5	1.11 ± 0.14	0.49 ± 0.13
pH 5.5 + 1 μM FA	0.66 ± 0.11	0.51 ± 0.21
pH 7.4	2.80 ± 0.52	0.03 ± 0.02
pH 7.4 + 1 μM L-LV	0.046 ± 0.01	0.01 ± 0.01

Values are means ± SEM of 3 separate experiments. FA indicates folic acid and L-LV, L-leucovorin. The uptake of MTX was measured at pH 5.5 with or without 1 µM FA (to inhibit PCFT-mediated uptake) and at pH 7.4 with or without 1 µM L-LV (to inhibit RFC-mediated uptake). The definition of uptake is pmol MTX per minute per 1 × 10^7^ cells.
